# Transmission Patterns of HIV and Hepatitis C Virus among Networks of People Who Inject Drugs

**DOI:** 10.1371/journal.pone.0022245

**Published:** 2011-07-20

**Authors:** Richard Pilon, Lynne Leonard, John Kim, Dominic Vallee, Emily De Rubeis, Ann M. Jolly, John Wylie, Linda Pelude, Paul Sandstrom

**Affiliations:** 1 National HIV and Retrovirology Laboratories, Public Health Agency of Canada, Ottawa, Ontario, Canada; 2 Epidemiology and Community Medicine, University of Ottawa, Ottawa, Ontario, Canada; 3 Centre for Communicable Diseases and Infection Control, Public Health Agency of Canada, Ottawa, Ontario, Canada; 4 Medical Microbiology and Community Health Sciences, University of Manitoba, Winnipeg, Manitoba, Canada; 5 Cadham Provincial Laboratory, Manitoba Health, Winnipeg, Manitoba, Canada; Food and Drug Administration, United States of America

## Abstract

**Background:**

The risk-related behaviours and practices associated with injection drug use remain a driver of HIV and hepatitis C virus (HCV) transmission throughout the world. Here we evaluated HIV and HCV transmission patterns in the context of social networks of injection drug users (IDU) recruited from a higher incidence region in order to better understand factors that contribute to ongoing transmission among IDU.

**Methods:**

IDU recruited through a chain-referral method provided biological specimens for analysis. HIV and HCV positive specimens were sequenced and analyzed using phylogenetic methods (Neighbour-joining and Bayesian) and transmission patterns of HIV and HCV evaluated in the context of the recruitment networks.

**Results:**

Among the 407 recruited IDU, HCV and HIV prevalence were 60.6% and 10.1%, respectively; 98% of HIV positive individuals were co-infected with HCV. Thirty-six percent of HCV sequences were associated with clusters, compared to 67% of HIV sequences. Four (16.7%) of the 24 HCV clusters contained membership separated by 2 or fewer recruitment cycles, compared to 10 (41.6%) derived from more than one recruitment component. Two (28.6%) of the 7 HIV clusters contained membership separated by 2 or fewer recruitment cycles while 6 (85.7%) were composed of inter component membership.

**Conclusions:**

Few HIV and HCV transmissions coincided with the recruitment networks, suggesting that they occurred in a different social context or a context not captured by the recruitment network. However, among the complete cohort, a higher degree of HIV clustering indicates many are recent infections originating from within current social networks, whereas a larger proportion of HCV infections may have occurred earlier in injecting history and in the context of a different social environment.

## Introduction

The risk-related behaviours and practices associated with injection drug use contribute to large numbers of infections with blood-borne pathogens [1], [2], [3]. In Canada, these practices are the largest contributor to new cases of hepatitis C virus (HCV) infections, as well as a significant contributor to the Canadian HIV epidemic, particularly among women [4]. Risk behaviors associated with injection drug use account for 17% of all new HIV infections and 70–80% of new HCV cases in Canada [5]. While national annual HCV incidence rates had declined to a nadir of 1.6 per 100,000 in 2006, more recent figures suggest a reversal in this trend with an incidence estimate of 2.2 per 100,000 in 2008. In 2005, Millson and colleagues [6] found that although HIV incidence among injection drug users (IDU) in the province of Ontario had remained relatively stable at 0.23 per 100 person years, the incidence within the city of Ottawa, Canada was 25 times higher at 5.8 per 100 person years. Knowledge of the distribution of these pathogens and of the social network interactions between IDU can combine to provide a better understanding of the dynamics of HIV and HCV transmission within this at risk population.

Located within the National Capital Region of Canada, the City of Ottawa consists of communities (neighbourhoods, suburbs, villages and rural areas) separated by both physical (distance, rivers, green space, highways) and demographic (language, socioeconomic, ethnicity/race) barriers. The most recent estimates (2002) suggest the number of people in Ottawa who inject drugs is 3,300, which equates to about 4 IDU per 1,000 general population [3]. Within the metropolitan area, IDUs tend to be concentrated in central core areas where health and prevention services have been made more readily available, although IDU populations are also present throughout the region with poorly characterized linkages to the central group and where access to services may be more limited.

In this study, members of IDU social networks in Ottawa were recruited using a chain referral method in order to identify and quantify social and sexual contexts that put IDU at risk of HIV and/or HCV infection. Since the intention is for the peers to be recruited from a study participant's personal social network, the recruitment chains represent social linkages that exist within the study population [7]. The method has proven to be effective at recruiting from populations that are hard to reach or small relative to the general population, and improving the chance of recruiting a more representative sample of the more marginalized, least accessible IDU who are farther removed from health and prevention services [8]. Biological specimens in the form of dried blood spots (DBS) were collected from all participants for serological HIV and HCV testing. For positive specimens, viral sequencing and phylogenetic analysis was used to establish the presence or absence of transmission linkages within the network. The purpose of this study was to document the impact of IDU social network structure on viral transmission dynamics so as to direct the development of prevention programs addressing individual risk practices and behaviours.

## Materials and Methods

### Recruitment of study participants

IDU from Ottawa, Ontario, Canada were recruited by their peers using a chain-referral method. Seven IDU ‘seeds’ who were selected by recruiters after participating in previous studies were recruited initially. In order to limit bias from highly networked individual, each was asked to recruit only three of their IDU contacts through the use of uniquely coded referral cards. Upon presenting for interview, each new recruit was also asked to refer three others in a similar fashion and so on, to a target enrolment of 400 participants. Research personnel kept track of who recruited who throughout the duration of the study. Potential participants were deemed eligible to participate in the study if they met all of the following criteria:

- Possession of a study recruitment card;
- Capable of providing informed consent;
- Injected drugs in the previous six months; and,
- Had not already participated in this study.

Participants completed a structured personal interview and provided a biological (DBS) specimen. The two part study instrument first collected demographic information including data on behaviours and practices documented to be associated with HIV and HCV infection, followed by questions addressing the investigation of the social and risk networks of IDUs based on standard egocentric methods of network analysis. All data and specimens were anonymous. All components of the study were approved by the Ottawa Hospital Research Ethics Board and reviewed and renewed on an annual basis. Subjects were informed of the purpose of the study and provided separate signed consent for the interview and for HIV and HCV blood tests.

Recruitment network diagrams were prepared using PAJEK software [9].

### Collection and storage of biological specimens

Dried blood spots (DBS) were collected onto 903 filter paper cards (Whatman, USA) by the standard finger prick method using a self-retracting lancet (Beckton-Dickinson, Canada). After saturating each of five printed circles, DBS were air dried for 3 hours (or over a weekend when collected on a Friday) at ambient temperature and humidity, and then stored in a vacuum desiccator at ambient temperature until weekly transport to the laboratory. DBS were placed in gas-impermeable zipper lock (Bitran Saranex) storage bags containing desiccant pouches for shipping and storage. At the laboratory, the desiccated DBS were stored at −80 C until testing was performed.

### Serological assays

DBS samples (¼ inch hole punch or equivalent) were screened for HIV using the Genetic Systems rLAV HIV-1 EIA (BioRad, Canada). Reactive samples having a signal to cutoff (sco) between 1.0–3.99 were repeated in duplicate. If both repeat wells were negative (sco<1.0), the sample was reported as negative. If either well was reactive (sco≥1.0) the sample went on to confirmatory testing using the Genetic Systems HIV-1 Western Blot (BioRad, Canada). Screened samples with sco≥4.0 went directly to Western Blot. Both of these assays are approved for use with DBS samples.

All DBS samples were also screened for HCV by eluting one ¼″ DBS punch into the Genetic Systems rLAV HIV-1 EIA diluent according to manufacturer's instructions and testing the eluates using the Ortho HCV Version 3.0 ELISA (Ortho-Clinical Diagnostics, Canada). Highly reactive samples (sco>2.0) were considered positive, low reactive samples (sco<2.0) were repeated in duplicate. Samples were considered reactive if either of the duplicates was reactive (sco>1.0). No confirmatory HCV assay was available at the time of testing.

All of the HIV and HCV serological assays used, including modification of the HCV assay, were validated in our laboratories for use with DBS.

### Molecular analysis

#### Isolation of Nucleic Acids from DBS

Nucleic acids were isolated from all HIV and/or HCV positive specimens for molecular analysis. Individual DBS were cut into 3–4 strips onto a clean sterile surface using sterile disposable Littauer suture scissors (Benlan Inc, Oakville, Canada). After eluting nucleic acids from DBS into 3 ml EasyMag Lysis buffer (buffered guanidium thiocyanate, BioMerieux Canada) for 1 hour at ambient temperature, viral nucleic acids were isolated from DBS eluates using the Nuclisens EasyMag system and reagents according to the manufacturer's instructions. Briefly, DBS eluates were transferred to EasyMag cassettes, magnetic silica added, and silica-bound nucleic acids washed to remove contaminants. In the final cycle, nucleic acids were eluted into a low-salt buffer [10].

#### Amplification & Sequencing

HIV and HCV genetic regions (2 each) were amplified from 10 µl nucleic acid extract using in house nested reverse transcriptase PCR (RT-PCR) methods. The HIV-1 primers and method used were designed to target all HIV-1 group M subtypes and are routinely used to determine strain and drug resistance in Canada. The method targets HIV pol, producing overlapping segments that span protease and the first 900 nucleotides of reverse transcriptase (RT). The method and primer sequences are available elsewhere [11]. The same protocol was used for HCV with primers that were developed to detect most genotypes found in Canada and have been used in several studies, but they have not been thoroughly validated against all genotypes. The HCV primers targeted portions of core (∼460nucleotides, 5′UTR.out.for: TGATAGGGTGCTTGCGAGTG; Core_A.out.rev: GAGAGCAGGGCCARIARGAAGATAGA; 5′UTR.in.for: GAGGTCTCGTAGACCGTGCA; Core.in.rev: GAGCAACCIGGIARRTTCCCTGTTG) and NS5B (550 nucleotides, NS5B-out.for: GTSTGGIARGACYTICTGGAAGAC; NS5B-out.rev: RGIGCRGARTACCTRGTCATAGCCT; NS5B-in.for: IACYATCATGGCIAARARYGAGGT; NS5B-in.rev: ACCTRGTCATAGCCTCCGTGAA). Amplified templates were sequenced in both the 5′ and 3′ orientations using an ABI Prism 3130xl genetic analyzer. Sequences were generated by aligning electropherograms to a reference sequence with SeqScape software (Applied Biosystems, Canada). Population-based (bulk) sequencing is the standard method used in the majority of molecular epidemiology studies of HIV and HCV. In the case of IDU, who are at higher risk of superinfection with HCV [12] and probably HIV, it is possible that superinfections may occur. Although the method used will miss clustering associated with minor superinfecting variants this will not detract from associations identified due to the majority species.

#### Phylogenetic Analysis

Sequences were aligned using Clustalx software [13]. Phylogenetic trees were generated using the neighbour-joining method of Saitou and Nei with the Kimura 2-Parameter model and pairwise deletion of gaps, as implemented in the MEGA 4.0 [14] software package. The reliability of the branching orders was assessed by bootstrap re-sampling (100 replicates). Cluster bootstrap values above 80% were considered significant. Subtype/genotype reference sequences were obtained from the HIV and HCV Sequence Databases (www.hiv.lanl.gov and www.hcv.lanl.gov).

## Results

### Study participants and networks

Four hundred and seven (407) IDU were recruited between September and December, 2007. All participants completed the interview process and only one refused to provide a DBS specimen.

**Figure 1:**
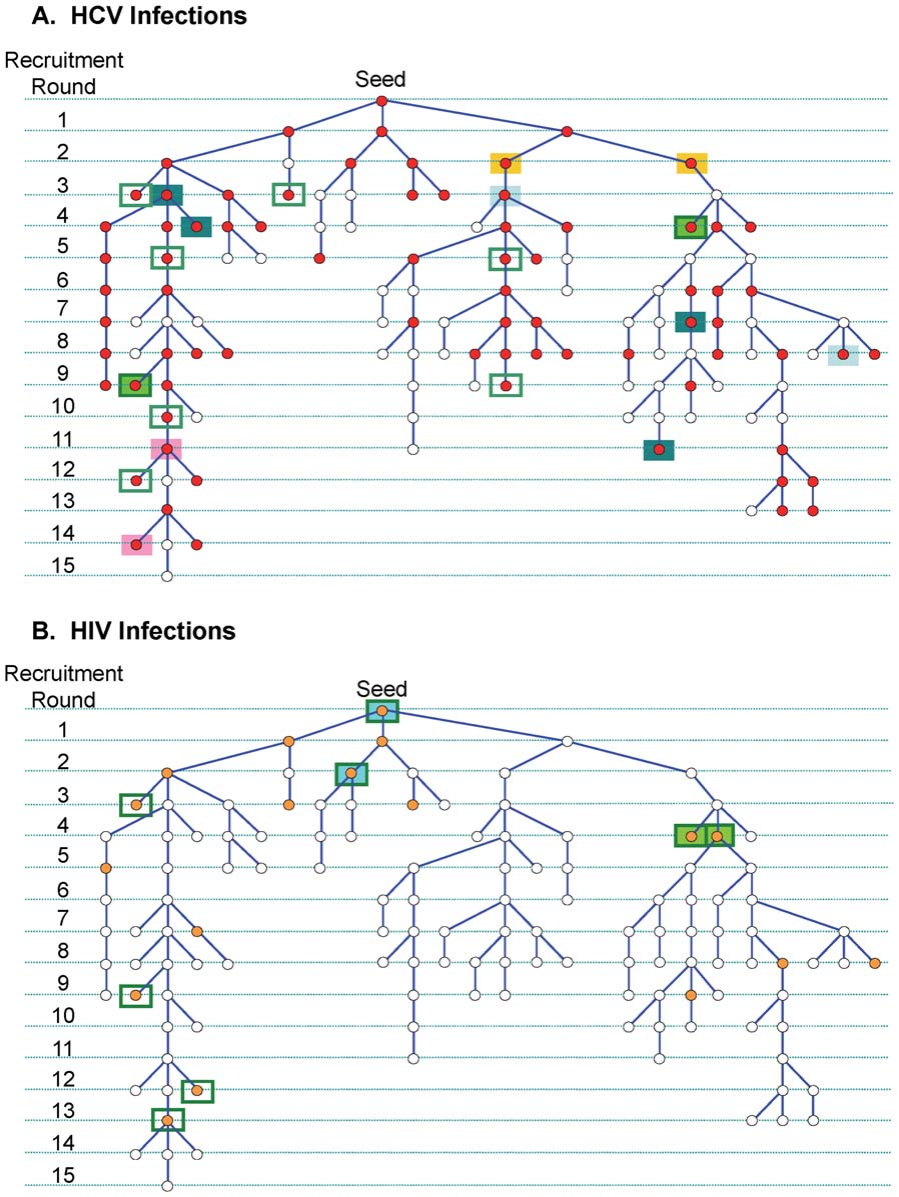
Recruitment network #2. Both **A** and **B** represent the same network of 126 participants. Red circles in A indicate HCV positive and orange circles in B indicate HIV positive participants. Coloured boxes indicate phylogenetically related infections within the network. Green frames indicate phylogenetic link(s) to other network(s) with (coloured) or without (white) links within the network. All branches have a length of 1. Vertical scale indicates recruitment rounds (distance) from seed.

As described in Table 1, the seven seeds generated four individual recruitment networks consisting of 12, 13, 126 and 253 members resulting from 4, 4, 15 and 22 recruitment rounds (chain lengths), respectively. Three seeds recruited no additional participants. The two largest networks represented 93% of the overall sample. Figure 1 is a schematic of the second largest recruitment network, showing recruitment rounds and relationships between HIV and HCV infections. The majority (80%) of the participants were male. The complete demographic data is presented as part of a separate manuscript.

**Table 1:** Characteristics of recruitment networks.

Network Component	Seed Gender	Size (n = )	Recruitment rounds	Male		Female		HCV positive	HIV positive
1	M	253	22	210	83.0%	43	17.0%	61%	9%
2	M	126	15	99	78.6%	27	21.4%	60%	14%
3	F	13	4	8	61.5%	5	38.5%	23%	0%
4	F	12	4	5	41.7%	7	58.3%	92%	0%
5	M	1	0	1	100.0%	0	0.0%	100%	0%
6	M	1	0	1	100.0%	0	0.0%	100%	0%
7	F	1	0	0	0.0%	1	100.0%	0%	0%
Total		407		324	79.6%	83	20.4%	60.6%	10.1%

### Serology

Of the 406 DBS specimens collected, 41 (10.1%) were confirmed anti-HIV-1 antibody positive and 246 (60.6%), anti-HCV antibody positive. All but one of the HIV positive specimens were also HCV positive, such that overall, 9.9% of the cohort was HIV/HCV coinfected. Among those HIV or HCV infected, the coinfection rate was 97.6% and 16.3%, respectively. One hundred and fifty nine (39.2%) specimens were negative for both HIV and HCV.

### Molecular Analysis of HIV and HCV

#### HCV genotyping / HIV subtyping

Only specimens that were HCV and/or HIV positive by serology were followed up with molecular analysis. Of these, we were able to generate sequences from at least one of the two genetic regions targeted for each virus from 74% (183/246) and 71% (29/41) of HCV and HIV positive specimens, respectively. In the case of HCV, the core and NS5B genetic regions generated 175 (71.1%) and 164 (66.7%) nucleotide sequences, respectively. In the case of two HIV positive specimens, only sequence from the RT genetic region was available.

HCV genotypes and HIV subtypes were determined by phylogenetic analysis performed with the inclusion of reference sequences. HCV genotypes (Table 2) were found to be predominantly 1a (64.5%) and 3a (21.9%). HCV genotypes for which sequence data was available from both genetic regions were in agreement for 152 of the 156 (97.4%) specimens. Three of the four discordant specimens were genotype 1a in core and 3a in NS5B, with the fourth being 3a in core and 2b in NS5B, possibly indicative of HCV superinfection.

**Table 2:** HCV genotype distribution.

Genotype	n =	%
1a	120	65.9
1b	9	4.9
2a	3	1.6
2b	9	4.9
3a	41	22.5
Total	182	100.0

As with HCV, the HIV subtypes were also determined using phylogenetic methods. Ninety percent (90%) of the HIV positive specimens were subtype B, with the remaining 10% subtype C. Subtypes were determined from *pol* (protease and RT) sequences except in the case of the two specimens for which only RT sequences were available.

Nucleotide sequences have been deposited in GenBank under the following accession numbers: HIV pol: JF817355–JF817383; HCV core: JF824151–JF824251 and JF824253–JF824326; HCV NS5B: JF824327–JF824490.

#### Clusters

**Figure 2:**
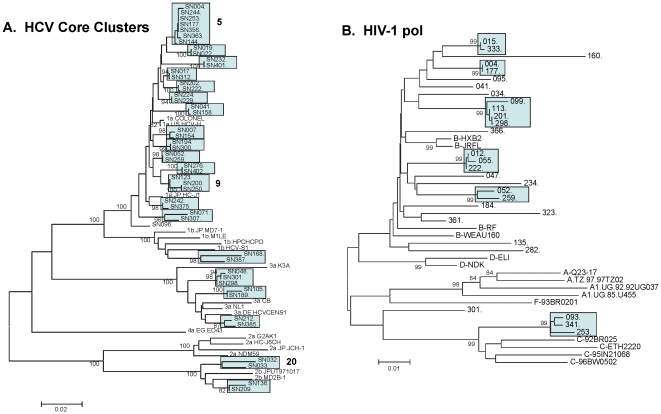
Phylogenetic analysis. **A**. Neighbour Joining analysis of all HIV-1 pol sequences generated. **B**. Analysis of all clustered HCV core sequences. In each tree, bootstrap values greater than 80% are indicated. In situations where HCV clusters based on core analysis (5, 9 & 20) fell below the 80% bootstrap cut off, similar analysis of NS5B (not shown) resolved clusters with bootstrap values above 80%.

Separate phylogenetic analysis of 176 core and 166 NS5B sequences revealed that 58 (32%) of the HCV specimens with amplifiable RNA were related to at least one other HCV infection within the recruitment networks. In all, 24 HCV infection clusters were identified in at least one of the two regions analyzed, with cluster size ranging from 2 to 7 participants [Figure 2A and Table 3]. Similar phylogenetic analysis of HIV sequences revealed that 18 (62%) of the 29 specimens for which HIV sequence data was obtained fell within 7 distinct transmission clusters ranging in size from 2 to 4 participants (Figure 2B). Of the 24 HCV clusters, only 4 (16.7%) contained membership separated by 2 or fewer recruitment cycles, in contrast to 10 (41.6%) clusters that were derived from more than one recruitment component. Two (28.6%) of the 7 unique HIV clusters, contained membership separated by 2 or fewer recruitment cycles while 6 clusters (85.7%) were composed of inter component membership.

**Table 3:** Composition of Phylogenetic Clusters and links within and between Network Components.

Virus	Cluster	Size	Intra Component LinksDegrees of Separation***	Inter Component Link(s)
HCV	1	3	0, 8, 9	
	2	2	3	
	3	2		YES
	4	2	2	
	5	7	2, 9, 11, 12, 20	YES
	6	2		YES
	7	2		YES
	8	2	15	
	9	3	0	YES
	10	3	2	YES
	11	2	5	
	12	3	8	YES
	13	2		YES
	14	2		YES
	15	2	0	
	16	2	6	
	17	2	3	
	18	2	8	
	19	2	1	
	20	2	16	
	21	2	2	
	22	2		YES
	23	3	11, 14, 14	
	24	2	4	
HIV	1	2	11	
	2	2		YES
	3	4	1, 20	YES
	4	3	1	YES
	5	2		YES
	6	2		YES
	7	3	9	YES

***number of recruitment cycles separating clustered specimens.

Among the 13 coinfected participants that were found to be HIV cluster members and where HCV sequence results were also available, 8 (61.5%) also demonstrated HCV clustering, including two cases of concordant clustering in which pairs of participants shared phylogenetically related HIV and HCV infections. Discordant clustering was observed in two cases of coinfection where the coinfecting viruses each clustered with separate members of the network. Conversely, of the 5 coinfected participants where HIV sequences were shown not to cluster and for which HCV sequence data was available, only 1 (20%) demonstrated clustering with an HCV infection of another network participant. Unfortunately in this case, although the related HCV infected partner was also HIV co-infected, we were unable to generate HIV sequence data thus pre-empting further cluster analysis.

## Discussion

Veiled risk behaviors and the marginalization of populations present unique challenges for understanding viral transmission dynamics within social networks of IDU. Here we have applied molecular phylogenetic analysis of HIV and HCV of infected participants collected through chain-referral sampling of Ottawa's IDU population, in an attempt to understand the impact of social network structure on the transmission of these viruses. The generation of unique genetic variants due to elevated replication rates and error prone reverse transcription allows genetic concordance to serve as a surrogate of epidemiologic linkages in the study of HIV and HCV transmission [15], [16], [17]. It is important to note that using these methods, it is not possible to determine the direction of transmission, or even if the virus was transmitted directly between contacts [18]. Rather we use phylogenetic clustering as an indicator of viral movement within the study populations in order to gauge the effect of social network structure on transmission dynamics.

By way of sampling hard-to-reach populations, chain-referral sampling methods, when coupled with phylogenetics should allow a unique perspective on viral transmission dynamics among IDU populations. It would seem reasonable that virus transmissions would occur more frequently between social contacts within a smaller network space (fewer recruitment steps between participants) than between those more distally related within a network or belonging to another network component altogether.

Perhaps the most surprising finding of this study is that viral transmission events do not appear to be occurring along the recruitment lineages of the network (dyads) or even within the immediate recruitment network space. For the purpose of this discussion, we define a transmission dyad as any two recruitment network direct contacts identified through the chain-referral sampling who are both infected with a phylogenetically related virus. Collectively, we have identified seven unique HIV phylogenetic clusters indicative of either indirect or direct intra/inter component transmissions. However, a common virus is never identified in any of the occasions where an HIV-positive participant recruits another HIV-positive contact, and only occurs in three instances for HCV infections. For the two occasions where two individuals share a related HIV infection and a common recruitment network contact (either uninfected or infected with a phylogenetically unrelated virus), the HIV cluster includes HIV positive participants recruited from within different components. Likewise only 10.3% of HCV phylogenetic clustering appears to have resulted from recruitment; in other words, an HCV-positive individual recruiting a contact infected with a phylogenetically related virus. In their study of 199 IDU, Aitken and colleagues found more of HCV clustering (18%) was due to social dyads, but one key difference between our studies is that the Aitken study only recruited partners with whom participants had injected in the previous 6 months [19]. In addition, through the use of named injecting ‘partners’, they found 577 dyadic relationships between participants, whereas we were limited to the 400 dyadic recruitment relationships from our 4 recruitment networks.

The question then becomes, what factors have resulted in such small numbers of transmission dyads being observed within the recruitment networks. It is important to note that the data presented in this paper represents only a snapshot in time of the social network structure of people in Ottawa who inject drugs and does not adequately describe the many possible (and probable) past and present social interactions between the recruited individuals. Two individuals who are only distally related within a single recruitment network component or reside within separate components at the time of recruitment may in fact be more closely related within the social network or may even currently exist as occult social contacts not captured as part of the recruitment process. Social interactions (links) between participants appearing distally related within the current recruitment network may be borne out by social network analysis based on questionnaire responses that will evaluate how closely any two individuals are truly related in the IDU social framework.

A limitation of this study is the potential bias of the sampling methodology. Targeting a difficult to reach population (IDU) can come at the expense of randomness. As such, additional analysis would be required in order to develop prevalence estimates of HIV/HCV for the IDU community as a whole. Future analysis will include an assessment of sampling bias and the effectiveness of the sampling methodology in reaching hidden populations.

As the majority of HIV and HCV positive individuals in this study reported prior knowledge of their infection status it is conceivable that a positive viral diagnosis coupled with public health-directed counseling has resulted in behavioral changes leading to social network restructuring. For example, information pertaining to safer injecting practices may have resulted in both the dissolution of an existing networked transmission dyad and the prevention of onward transmission thus limiting the formation of additional dyads with new social contacts. An understanding of how long recruitment network participants (infected or not) knew each other might shed light on how the durability of network connections influences transmission patterns.

This said, it is also possible that network structure may be more or less stable and that clustering in the absence of transmission dyad formation may be an indication of hidden epidemic drivers. It is possible that the recruitment process utilized did not sufficiently sample from the most marginalized portions of the IDU network, which by default would also be those with the least access to public health interventions. It may be the hidden portions of the network that serve as transmission bridges between distal portions of the larger components or between independent components. Intuitively, we would likely have seen the imprinting of network recruitment on phylogenetic clustering if HIV or HCV outbreaks were occurring within portions of the visible network.

In the case of HCV, with an overall network prevalence of 60.6%, it may also be difficult for the determinants of transmission dyad formation (risk behavior and serodiscordance among current social network contacts) to occur simultaneously. While the likelihood of contact with an HCV-positive individual is elevated, the most susceptible IDU (most at risk by behavior) would have been exposed and infected very early in their injecting history [20], [21] thus preventing the formation of a transmission dyad, and leading to the relatively low level (38%) of HCV phylogenetic clustering observed in the study. Possibly the herd immunity effect imposed by prevalent HCV infections coupled with effective public health interventions tied to HIV prevention, disrupts the transmission chains within the visible network, although transmissions may continue to flow along undetected and otherwise unsampled portions of the IDU network.

While the HCV infections detected may have occurred earlier in participants' injecting histories, and in the context of a different social network structure, the opposite may be true for HIV infections. Although the prevalence of HIV remains low at 10.1%, a large percentage of these infections formed clusters within the recruitment networks (62.1%), suggesting that many of the HIV infections are more recent, with their origin residing in the context of the current social network.

This study demonstrates that the complementary use of social network analysis and molecular epidemiological methods can be effective in describing the transmission dynamics of blood-borne pathogens among persons who inject drugs, information that is essential to targeted prevention strategies and sound public health policy.
